# Oxygenated-Blood Colour Change Thresholds for Perceived Facial Redness, Health, and Attractiveness

**DOI:** 10.1371/journal.pone.0017859

**Published:** 2011-03-23

**Authors:** Daniel E. Re, Ross D. Whitehead, Dengke Xiao, David I. Perrett

**Affiliations:** School of Psychology, University of St Andrews, St Andrews, United Kingdom; Nothwestern University, United States of America

## Abstract

Blood oxygenation level is associated with cardiovascular fitness, and raising oxygenated blood colouration in human faces increases perceived health. The current study used a two-alternative forced choice (2AFC) psychophysics design to quantify the oxygenated blood colour (redness) change threshold required to affect perception of facial colour, health and attractiveness. Detection thresholds for colour judgments were lower than those for health and attractiveness, which did not differ. The results suggest redness preferences do not reflect a sensory bias, rather preferences may be based on accurate indications of health status. Furthermore, results suggest perceived health and attractiveness may be perceptually equivalent when they are assessed based on facial redness. Appearance-based motivation for lifestyle change can be effective; thus future studies could assess the degree to which cardiovascular fitness increases face redness and could quantify changes in aerobic exercise needed to increase facial attractiveness.

## Introduction

Red colouration of the skin affects group interactions in several primate species. Redness levels in the faces of male mandrills correlate with position in the dominance hierarchy, with intensely coloured males gaining higher positions [1], [2]. Male macaques experience a rise in testosterone during mating season [3] which increases redness of the face [4], making the faces more preferable to females [5]. Red skin colouration is also present in some female primates, and acts as a signal of fertility. The hindquarter region of female macaques shows increased red colouration during periods of high fertility, and males selectively attend to this signal [6]. The intensity of facial redness fluctuates in female mandrills, with faces being brightest red while fertile [7]. Female rhesus macaques also selectively attend to red faces and hindquarters of other females, suggesting red colouration may be salient to intrasexual interaction [8]. Male chacma baboons show increased sexual arousal when presented with red female perineum, the only colour to elicit such a response [9]. It has been suggested that red signals in primates are of such importance that they may have driven the evolution of the primate red-green colour system [10].

Red colouration seems to affect human interactions. Hill and Barton [11] studied Olympic competitions in physical combat and found that, when controlled for skill level, athletes wearing red were more likely to win than those wearing blue, even though colours are arbitrarily assigned. In virtual simulations, football goalkeepers are less confident in stopping penalty kicks from players wearing red [12]. Tae kwon do referees are more likely to award points to combatants wearing red, even if their performance is identical to an opponent wearing blue [13]. Facial flushing when angry increases redness in both men and women [14], [15], and red faces are more likely to be perceived as angry and dominant than fearful [16]. Indeed, even in simplistic shapes such as circles, red is perceived as more aggressive, dominant, and more likely to win in physical competitions over blue [17].

Despite its association with aggression and dominance, red has a well-documented association with human sexuality. Red is associated with love and romance across several cultures and age groups [18], [19]. Anthropologists suggest that red ochre was used as body and face paint on women in ancient civilisations, possibly to symbolise sexual fertility [20]. Similar to females of other primate species, displays of red (such as red clothing) make women appear more attractive to men [21]. Likewise, displays of red on men increase women's perception of men's status and overall attractiveness [22]. Women have been using red ornamentation on their lips to heighten their attractiveness since 10,000 B.C. [23]. Human sexual excitation is often accompanied by red flushing of the skin that spreads from the chest to the neck and face [24]. These findings suggest that, as in other primate species, red colouration is associated with human sexual attraction.

In humans, high levels of oxygenated blood causes bright red colouration of the skin [25]. High blood oxygenation is indicative of cardiovascular fitness and can be increased with aerobic exercise [26]. In women, higher estrogen levels are associated with increased vascularisation [27] and increased vasodilation [28], two responses that increase arterialisation of blood in the skin [29]. Conversely, low blood perfusion to the skin causes visible skin pallor and is associated with diseases like anaemia [30]. High levels of deoxygenated blood cause a bluish tint in the skin, which may indicate coronary or respiratory illness [31].

Stephen, Coetzee, Law-Smith and Perrett [32] tested whether manipulations of facial redness (simulating changes in levels of oxygenated blood) affected perception of health. They found that increasing skin colouration associated with raised blood oxygenation increased perceived healthiness in 98% of young adult Caucasian faces tested. When able to simultaneously manipulate skin colour along oxygenated and deoxygenated blood colour axes, participants chose to increase oxygenated blood colour and decrease deoxygenated blood colour. Furthermore, the amount of oxygenated blood colour added to faces negatively correlated with initial colour of the face; thus the lower the face redness was to start with, the more oxygenated blood colour that was added to optimise the appearance of health.

While increasing oxygenated blood colouration (henceforth referred to as redness) produces healthier-looking faces, the change in colour needed for this effect to be perceptible is unknown. It is possible that human preferences for redness may reflect a sensory bias, in which case any perceptible change in red colour could affect perceived health or attractiveness. Colour preferences in mate choice have evolved from non-sexually based sensory biases in several species [33], [34]. For example, food colour preference may have led to mating colour preferences in guppies [35] and bowerbirds [36]. Sexual preferences for red colouration may have been shaped by red food preferences in three- and nine-spined sticklebacks, and do not reflect a learned colour preference [37]. Humans may have a similar bias towards red. Indeed, human preferences for the colour red are found in infants at 4–5 months of age [38], and British and Chinese women show preferences for reddish contrasts on white backgrounds [39]. If human preferences for facial redness stemmed from a pre-existing (unlearned) sensory bias towards red, any perceptible change in redness could alter perceived facial attractiveness (assuming all other facial features are held constant). Conversely, redness preferences may reflect mate choice decisions based on indications of underlying physiological fitness or reproductive hormonal state. If this was true, and assuming all other face features were held constant, the colour change needed to alter perceived health and attractiveness may exceed the threshold for simple colour discrimination. A reliable change in fitness or hormonal status may only be indicated by a substantial change in skin blood perfusion or oxygenation. Determining psychophysical thresholds for redness discrimination and health and attractiveness judgments may help determine the basis of preferences for facial redness.

Facial attractiveness is thought to signal underlying health [40], [41], and several features of face attractiveness (such as symmetry, averageness, and dimorphism) have little effect on attractiveness when perceived health is statistically controlled [42], [43]. However, what is perceived as optimally healthy and what is perceived as optimally attractive do not always align in human face stimuli [44]. It is therefore possible that minor fluctuations in facial redness may be enough to alter perceived attractiveness, whereas perception of health can only be altered by much more prominent redness differences (or vice versa). Determining psychophysical thresholds for facial redness in health and attractiveness judgments will reveal the perceptual association between health and attractiveness when assessed based on facial redness.

Facial redness is a result of blood oxygenation and skin perfusion [25], which can be augmented through aerobic training [26]. Determining the redness change thresholds for perceived facial health and attractiveness may allow researchers to quantify the weekly amount of aerobic exercise required to become healthier-looking and more attractive. Previous research finds that demonstrating the improvement in appearance resulting from a healthier lifestyle can motivate healthier living [45]. Quantifying the exercise enhancement needed to become more healthy-looking and attractive may lead to more ambitious and goal-driven exercise regimens.

In the current study, we manipulated facial redness to simulate changes in blood oxygenation level. We then determined redness change thresholds needed to alter three perceptual parameters: facial redness, health, and attractiveness. The results of this study could help determine the nature of redness preferences, and could be used to quantify the increases in aerobic exercise needed to augment perceived facial health and attractiveness.

## Methods

The study was performed under the approval of the University of St Andrews ethics committee. All participants gave written informed consent prior to participating.

### Skin pigmentation determination

We used the methods of Stephen et al. [32] to create accurate oxygenated blood colouration. Spectrophotometer measurements (specular component excluded, illuminant d65, 10° observer angle) were taken of the first dorsal interosseous region of the left hand, in CIE L*a*b* space, after 2 minutes of the hand resting on a desk. We then took further measurements after 5 minutes of the hand resting in hot (45–50°C) water, which causes a high degree of hyperaemia and increases blood arterialisation. Skin colour changes associated with oxygenated blood levels are similar between the hand and face [32].

### Photography

Photography conditions were identical to those in Stephen et al. [32]. Two men and 2 women (mean age  = 20, range  = 19–21) were photographed with neutral expressions, without make-up in a light-controlled photo booth. Participants were asked to remove spectacles and visible jewellery, and maintain a neutral expression. Photographs were taken using a Fujifilm FinePix S5Pro digital SLR camera with a fixed-length 60 mm lens in a booth painted on all surfaces with Munsell N5 grey. Illumination was exclusively provided by three Verivide 6504K daylight simulation bulbs. Participants held a Munsell N5 painted board over their shoulders to obscure reflections from clothing. A GretagMacbeth Mini ColourChecker chart was included in each image in order to colour-calibrate images. Images were colour-corrected by transforming observed RGB values of each of the 24 colour-checker patches towards spectrophotometer-determined CIE L*a*b* values of these same patches using a least-squares transform from an 11-expression polynomial expansion [46]. This resulted in a mean colour-error (ΔE) of 1.97 where ΔE is a standard means of presenting colour-error in the CIE L*a*b* colour space and here represents the Euclidean distance between calibrated image and reference colour patches.

### Image manipulation

**Figure 1:**
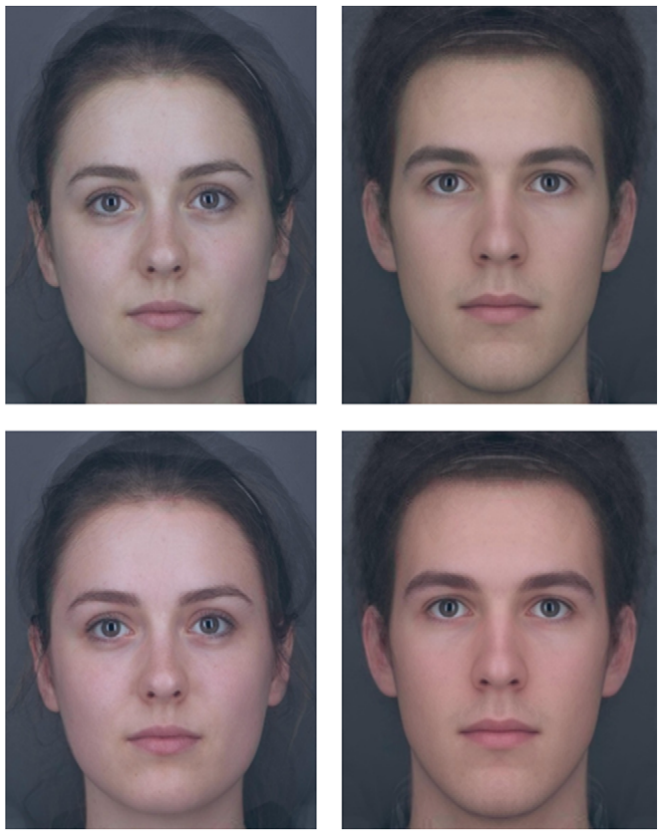
Examples of the type of face stimuli used. The top row shows the lowest oxygenated blood colour of the stimuli tested, while the bottom row shows the highest. The colour difference between the top and bottom rows is 4.8 (ΔE). These images were averaged from several faces and were not used in the actual experiment, but accurately represent how faces were presented and show the colour difference at the extreme ends of the colour continuums.

Two face colour masks were created in Matlab, one simulating the high blood oxygen pigmentation, and one simulating the low blood oxygen pigmentation. The ΔE between the two masks was 2.4. A colour continuum of 64 images ranging from the low to the high oxygen pigmentation colour was created by applying ±100% of the colour difference between the two masks to all skin portions of four unmanipulated face images (2 men, 2 women; see Figure 1 for an example of the type of stimuli used). This created a continuum of faces where the extreme ends had a ΔE of 4.8. The hair, eyes, and background of the images remained unmanipulated. All face images were presented at the same size on a colour-calibrated CRT screen. Participants sat approximately 50 cm away from the screen in a darkened booth.

### Participants and procedure

Twenty-six undergraduate students (*mean age*: 18.9, *SD*: 0.95, *range*: 18–22; 7 males, 19 females) participated in the study for course credit. All participants reported having normal colour vision. All 26 participants were included in the analysis for redness discrimination; only 24 participants were included in the analysis of the health and attractiveness tasks due to experimenter error (one participant missed both tasks; two other participants missed one task each).

Participants completed a 2-alternative forced-choice (2AFC) task that was divided into three blocks: redness, health, and attractiveness discrimination. The task ran as follows: a face was displayed for 750 ms, followed by a brief (100 ms) visual mask of random black dots (covering approximately half of the visual area, each dot was a few pixels) on a white background, followed by the same face with a different degree of redness displayed for 750 ms. Participants were then asked which face (the first or second) looked more red (redness discrimination), healthy-looking (health discrimination), or attractive (attractiveness discrimination). The order of presentation of the three blocks were randomised, and there were no biases toward any specific presentation order by chance.

Previous work has demonstrated that oxygenated blood colour makes faces appear healthier [32], and facial attractiveness is often based on cues of health [42]. We therefore assumed that higher facial redness would be perceived as more healthy and attractive in this experiment. Within each block, redness change thresholds were determined for each of the four faces using a staircase method design. Each block started with trials displaying faces at the extreme ends of their colour continuum (ΔE = 4.8). If participants chose the face with higher redness, the next trial using that face would have half the colour difference of the previous trial (e.g. – if the redder face was selected in the first trial, the second trial using that face would display a ΔE of 2.4). The mean colour of the two faces in each trial remained constant throughout the staircase. If the participant chose the face with lower redness in the first trial, the trial would be repeated; if they chose the face with lower redness four consecutive times, the colour threshold would be recorded as 4.8 (the colour difference at the extreme ends of the continuum). If the participant chose the face with higher redness in the trial on the staircase with the smallest colour difference (if they got that far), the trial was repeated. If they chose the face with higher redness four consecutive times, the colour threshold would be recorded as 0.075 (the colour difference between each interval in the continuum). The four faces were interleaved within each block so consecutive trials would rarely display the same face. This was done to avoid visual adaptation to a particular face, which could affect redness change thresholds. The program established redness change thresholds for each of the four faces separately; that is, trials displaying one face would not affect the ‘staircase’ status of the other faces. If participants chose the face with lower redness, this would constitute a staircase ‘reversal’, and the next trial with that face would have double the colour difference of the previous trial. Threshold values were defined as the average colour difference of three staircase reversals. Staircase reversals were only counted after the participant chose the face with the higher redness in the first trial.

## Results

Thresholds were analysed for the four faces for each judgment type. A repeated-measures ANOVA found no significant differences between faces in tasks for redness discrimination (*F*(3, 75) = 2.23, *p* = 0.09, η^2^ = 0.08), health (*F*(3, 69) = 0.62, *p* = 0.61, η^2^ = 0.03) or attractiveness (*F*(3, 69) = 0.62, *p* = 0.60, η^2^ = 0.03). We therefore collapsed thresholds across faces to calculate average thresholds in each of the three judgment types.

**Figure 2:**
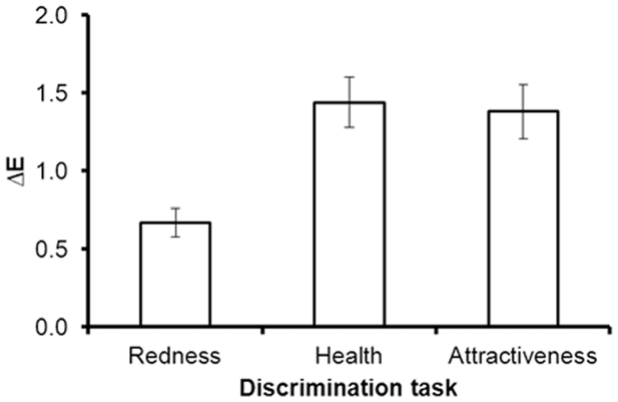
Discrimination thresholds and standard error in terms of ΔE values for redness, health, and attractiveness discrimination.

The average ΔE threshold was 0.67 (*SEM*: 0.09) for redness discrimination, 1.44 (*SEM*: 0.16) for health discrimination, and 1.38 (*SEM*: 0.17) for attractiveness discrimination (Figure 2). There was no effect of participant sex on thresholds in the redness (F(1,24) = 1.30, p = 0.27, η^2^ = 0.05), health (F(1,22) = 0.08, p = 0.78, η^2^<0.01), or attractiveness (F(1,22) = 0.37, p = 0.55, η^2^ = 0.02) discrimination thresholds. There were no significant interactions between face sex and participant sex in the redness (F(1,24) = 0.37, p = 0.54, η^2^ = 0.02), health (F(1,22) = 0.62, p = 0.44, η^2^ = 0.03), or attractiveness (F(1, 22) = 0.40, p = 0.53, η^2^ = 0.02) discrimination thresholds.

Paired-samples t-tests revealed that thresholds for redness discrimination were significantly lower than those for health (*t*(23) = −3.97, *p*<0.01, Cohen's d = −0.84) and attractiveness (*t*(23) = −3.64, *p*<0.01, Cohen's d = −0.78). Thresholds for health and attractiveness were not significantly different (*t*(22) = 3.92, *p* = 0.70, Cohen's d = 0.08).

## Discussion

Though face colour is just one of several cues to health and attractiveness, the current experiment saw manipulations only in facial redness. Thus, any differences in perceived health and attractiveness in this study came from changes in facial redness. If human redness preferences resulted from a sensory bias, a detectable difference in redness between two otherwise-identical faces should alter their perceived attractiveness. The results of this study show that a small but detectable change in redness does not necessarily alter perceived facial health and attractiveness when using the same testing procedures. Our results are consistent with an attraction to redness that reflects innate or learned preferences for reliable colour cues to health and mate choice, for example blood oxygenation levels [25] associated with cardiovascular fitness [26]. Skin redness increases with oestrogen levels in women [27], which are raised during periods of high fertility [47]. Redness in women may therefore not only indicate cardiovascular fitness, but may also be a subtle cue to fertility, as is found in females of other primate species [6], [7]. Redness change thresholds for the health and attractiveness tasks were not significantly different. One possible interpretation of this finding is that health and attractiveness are perceptually equivalent when they are assessed based on facial redness. Skin redness is indicative of blood oxygenation [25], and thus cardiovascular fitness [26]. It is conceivable that facial redness preferences may have evolved to exploit this cue to health. Differences can arise between perceived optimal health and attractiveness in some facial parameters, such as facial adiposity [44] but similar redness change thresholds for perceived health and attractiveness suggest that the two attributions are closely linked for colour. Facial redness may be another parameter where attractiveness is linked to underlying health [41]–[43].

The redness discrimination task required a smaller redness change than did the health or attractiveness tasks. It is possible that these threshold differences can be attributed to differences in the degree of neural processing. The redness discrimination task requires a straightforward decision on colour intensity [48]–[49]. Judgments on health and attractiveness are more complex and likely to require involvement from reward centres of the brain before judgments can be made [50]–[54]. It is possible that the threshold differences found in the current study are due to an extra step in processing the health and attractiveness judgments. Other psychophysical studies on social attributions, however, have found that stimulus change thresholds for social attributions are not always higher than those for simple discrimination. For example, thresholds for voice pitch discrimination and attribution of vocal masculinity (a trait associated with pitch) are not significantly different (Re, unpublished data). It is therefore conceivable that threshold differences between stimuli discrimination tasks and social attribution tasks depend on how closely linked the social attribution is to the sensory cue. For example, threshold differences between redness discrimination and health and attractiveness judgments are likely to be smaller than the threshold differences for a cue less well associated with health and attractiveness.

The current experiment used two men's and two women's faces to establish thresholds for facial redness in assessing health and attractiveness. Whereas thresholds did not differ between these four faces, we cannot be sure that thresholds would not differ for faces that are initially very low or high in perceived health and attractiveness. For example, a face that is already very healthy-looking and attractive may need a greater change in redness to increase perceived health and attractiveness. Increasing redness raised perceived health and attractiveness for each of the four faces used here (otherwise the staircase procedure used would not work), and this indicates there were no ceiling effects for perceived health and attractiveness for these four faces. Further research could determine whether or not thresholds depend on initial perceived health and attractiveness.

It is important to note that facial redness is not only associated with blood oxygenation [25] and oestrogen levels [27] but also with emotions such as anger and embarrassment [14], [15]. Fluctuations in skin redness in response to such emotions are transient, however, and pass relatively quickly [14]. Long-term preferences for facial redness are unlikely to reflect such short-term fluctuations, and more likely stem from stable long-term cues to physiology (though short-term increases in skin redness brought on by sexual excitement may also be a cause of attraction).

It should be noted that the current experiment presented trial images sequentially, for a short (750 ms) period of time, with a visual mask (100 ms) presented in between. This was done to avoid visual adaptation to faces, and is obviously not how faces are naturally viewed. Different methods of image presentation, such as presenting faces for longer or without the intervening mask, may yield lower thresholds. Nonetheless, the divergence of thresholds for redness detection and judgments of health and attractiveness is evident with our methods.

Skin redness, associated with oxygenated blood levels, is enhanced with aerobic training [26]. The results of this study quantify the colour change needed to increase perceived attractiveness. Future research could quantify how changes in aerobic exercise (i.e., number of hours exercising per week) affect skin redness. Such research could then describe the changes in exercise regimen needed to produce a noticeable change in facial health and attractiveness. The ability to quantify how much of an improvement in cardiovascular fitness is needed to increase facial attractiveness could lead to more goal-directed exercise regimens and may add incentive to practise healthier lifestyles.
